# Long noncoding RNA MST1P2 promotes cervical cancer progression by sponging with microRNA miR-133b

**DOI:** 10.1080/21655979.2021.1921550

**Published:** 2021-05-25

**Authors:** Rongrong Xu, Xiaoyue Zhang, Yan Xu, Junqin Wang, Zhihui Li, Xiaoming Cui

**Affiliations:** Department of Obstetrics and Gynecology, Dongtai Traditional Chinese Medicine Hospital, Dongtai City, Jiangsu Province, China

**Keywords:** LncRNA MST1P2, miR-133b, cervical cancer, progression, metastasis

## Abstract

Long noncoding RNA (lnc RNA) is aberrant expressed in many kinds of tumors and may be concerned with the occurrence and progression of tumors. Lnc RNA MST1P2 is increased in cervical cancer (CC), but its mechanism in CC has not been clarified. In this study, RT-qPCR was employed to analyze Lnc MST1P2 and miR-133b expression. CCK8 and cell apoptosis assay detect the proliferation optical density (OD) value and apoptosis rate. Cell metastasis was evaluated by Wound-healing assay and Transwell assay. Dual-Luciferase assay analyzed the relationship between Lnc MST1P2 and miR-133b. In vivo experiment was performed by establishing xenograft animal model. We found that Lnc MST1P2 is obviously overexpression in CC tissues and cells. Si-Lnc MST1P2 obviously repressed cell growth, cell migration, and cell invasion in Hela and SIHA cells. Moreover, Si-Lnc MST1P2 suppressed CC tumorigenesis in vivo. Dual-Luciferase assay and RT-qPCR assay further proved that Lnc MST1P2 has a negative regulation to miR-133b. miR-133b up-regulation inhibited cell viability and metastasis of Hela and SIHA cells. miR-133b inhibition notably decreased the anti-cancer effect of si-Lnc MST1P2. LncRNA MST1P2 serves as a Cervical Cancer oncogene by sponging with miR-133b.

## Graphical abstract

**Figure:**
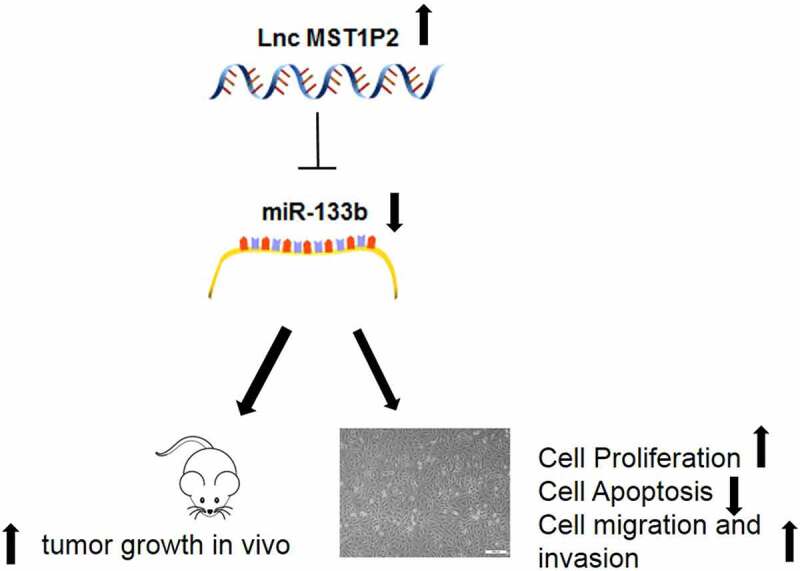

## Introduction

Cervical cancer (CC) is a common malignant tumor in women. The incidence rate and mortality rate of cervical cancer are fourth in women [1]. According to statistics, there are about 500,000 new cases every year, occurs in developing countries mostly [2]. In china, number of new cases per year accounts for about one third of the total number in the world [3]. Although the development of human papillomavirus (HPV) DNA detection and the standardized application of chemoradiotherapy technology recently have reduced the incidence and mortality of CC to a certain extent, the recrudescence rate of CC is still as high as 40%, and the mortality rate in developing countries is as high as 87% [4,5]. Therefore, understanding the pathogenesis of cervical cancer, looking for potential prognostic and targeted treatment indicators is of great significance to improve the subsistence rate of patients with CC.

Long noncoding RNA (lncRNA) usually refers to RNA transcripts (length: over 200 nucleotides), and dont have protein coding function [6]. In recent years, the research of lncRNA has attracted people’s attention. Numerous research have shown that lnc RNA could serve as competing endogenous RNAs via sponging of miRNAs is involved in chromatin modification, transcriptional regulation, epigenetics, post transcriptional processing and nuclear translocation [7–10]. It also plays a crucial role in CC development [11]. Study have shown that lncRNA XLOC_006390 could play a crucial role in CC malignancy by modulating miR-331-3p and miR-338-3p [12]. LncRNA SNHG1 and LncRNA SNHG7 was demonstrated to be up regulated and to induce cell growth and metastasis in CC [13,14]. lncRNA MST1P2 (lnc-MST1P2) was confirmed obviously up-regulated in DDP-resistant bladder cancer cells. lncRNA MST1P2 enhanced the drug-resistance of bladder cancer to cisplatin via regulating miR-133b mediated Sirt1/p53 signaling pathway [15]. Here, lncRNA MST1P2 was found significantly increased in CC compared with normal, however, the underlying mechanism of role of lncRNA MST1P2 in CC was unknown.

MicroRNA (miRNA) is small RNA molecules which have no protein coding function and post transcriptionally mediates the expression of target genes. MiRNA abnormal expression have been confirmed is correlated with many types of human malignant tumors, including CC [16–18]. It has been shown that the expression of miR-133b was down-regulation in breast cancer and induce cell metastasis [19]. miR-133b Inhibition suggests a bad prognosis of Oral squamous cell carcinoma [20]. It has been shown that MicroRNA-133b was down-regulated and could serve as a novel target in cervical carcinoma development [21].

This study aims to explore the expression of lncRNA MST1P2 and miR-133b in CC, the molecular mechanism in CC cells and the function in nude mice transplanted tumor, to lay a theoretical foundation for possible clinical application in the future.

## 1. Materials and methods

### 1.1 Subjects

The inclusion criteria of CC patients were based on pathological examination. All cervical cancer samples were confirmed by histopathology or cytology. All subjects signed informed consent. This study was approved by ethics committee.

### 1.2 Cell culture and cell transfection

CC cell lines (HeLa, C-33A and SIHA) and normal cervical epithelial cells were obtained. The cells were grown in DMEM medium containing 10% FBS at 37°C, 5% CO2 and saturated humidity.

The knockdown of Inc MST1P2 in cells was carried out by transfecting with Si-LNC- MST1P2. MiR-133b was overexpression or down-regulation by miR-133b or miR-133b-inh. The whole transfection using lipo3000 (Invitrogen).

### 1.3 RT-qPCR

Using Trizol extract RNAs. 200 μ l trichloromethane was added to accelerate the separation of RNA phase. In this step, shaken the EP tube 15 s, centrifuged at 12 000 rpm/min for 10 min. Take 400 μ l of supernatant liquid, add equal volume of isopropanol, refrigerate overnight at – 20°C. The precipitate was washed with 75% ethanol, centrifugation for 5 minutes at 8 000 rpm/min and dried at room temperature. The precipitates were dissolved. The first strand of cDNA was produced accordance with the instructions of reverse transcriptase m-mlv for first strand cDNA kit. Using the above cDNA as template, SYBR Premix ex taqtm Kit (Takara) was used in ABI VIIa 7 fluorescent real-time quantitative PCR (Applied Biosystems) was used for detection. The internal reference using GAPDH. The 2− ΔΔCt method was used for gene expression quantification.

### 1.4 Cell viability analysis

The medium containing 10% fetal bovine serum was used to prepare cell suspension with suitable concentration. The cells were added into 96-plates with 5000 cells per well. Six parallel wells were set up and cultured for 48 h at 37 °C and 5% CO2. The OD at 450 nm was recorded by CCK8 assay.

### 1.5 Cell apoptosis assay

Cells were carefully digested into single cell suspension, put into 1.5 ml EP tube, centrifugation at 1 000 g for 5 minutes. The cells were washed once with ice precooled PBS, then resuspended in 70% ethanol and fixed overnight at –20 °C. Centrifugation for 1 000 g for 5 minutes. Discard ethanol, wash once with ice precooled PBS, centrifuge, discard PBS, add PI dye solution to final concentration of 50 μg/ml, TritonX-100 final concentration of 0.25% (volume ratio) and RNase with final concentration of 100 μg/ml. The apoptosis rate was then evaluated by flow cytometer.

### 1.6 Cell migration and invasion assay

Wound-healing test to analyze ability of cell migration. Cells were added in 6-well plates and cultured to 90% concentration. Using a 200 μl pipette to make artificial wound.

The cell invasion assay was performed by chamber marigel invasion 24 well, BD BioCoat. Matrigel was diluted with FBS-free DMEM medium (1: 8) and acceded into the chamber. After 1 h, the diluent was discarded and cleaned twice with DMEM medium preheated at 37°C for invasion test. Cells were resuspended. In Transwell, DMEM including 10% FBS was accede to the lower chamber, and 200 μl DMEM was accede to the upper chamber (8 × 10^4^ cells/well). Culture at 37 °C for 12–24 h. Take out the chamber carefully, absorb the liquid from the upper layer of the chamber, scrub the upper layer of the chamber for 5 times to prevent deformation of polycarbonate membrane, and fix it with ice pre-cooled methanol for 20 minutes. Add 0.5% crystal violet dye solution into 24 well plate, 600 μl per well. Take out the fixed chamber from methanol, cotton swab wipes the upper layer of the chamber, and then put it into crystal violet staining solution for 10 minutes. Take out the chamber, wash the chamber with running water for several times, cotton swab wipes the upper layer of the chamber and the cells that have not been wiped clean, dry the film with air, place the film on the slide with the bottom of the film facing up, and take photos and count under the microscope from five randomly selected fields.

### 1.7 Western blot analysis

RIPA lysate was added into the cell to extract the total protein, SDS polyacrylamide gel was prepared and electrophoresed. The electrophoresis was performed at 80 V for 30 min, and then the voltage was adjusted to 120 v. After electrophoresis for 1 h, transferred the protein to PVDF. Then put it in the blocking solution and incubate for 1 hours. Added first antibody and incubated at 4 °C for overnight. The PVDF membrane was washed for 10 min × 3 times. Then add the second antibody (1: 2 000 dilution) and incubate at room temperature for 1.5 h. Enhanced chemiluminescence (ECL) solution was used to protein develop. The protein expression was detected by ImageJ. GAPDH as an internal control.

### 1.8 Dual-luciferase reporter analysis

Co-transfected Lnc MST1P2 and miR-133b, NC-inh, miR-133b-inh, with pmirGlo-NC, pmirGlo-Lnc MST1P2-mut or pmirGloLnc MST1P2-wt into HeLa and SIHA cells. The luciferase activity was measured at least three times by Dual-luciferase activity analysis system (Promega) at 48 hours after transfection.

### 1.9 Immunohistochemistry analysis

Sections were dewaxed in xylene, and then recycled with primary alcohol and antigen water in turn. The samples were boiled at 95 °C for 10 minutes under microwave irradiation, and then dissolved in EDTA. The biopsy samples were boiled in 3% hydrogen peroxide for 30 min, and then sealed with 20% goat serum for 40 min. The primary antibody was cultured in serum (1:100) at 4 °C overnight. All sections were cultured with HRP binding protein secondary antibody for 60 min, stained with 2-aminobenzidine, and stained with hematoxylin.

### 1.10 In vivo tumorigenesis assay

24 male nude mice (BALB/c-nu, 4–5 weeks) was purchased and propagated under special pathogen-free (SPF) conditions, subcutaneous injected Hela cells into the left armpit of mice. Tumor volume and tumor weight were evaluated every 4 days, all mice were sacrificed at the end, tumor tissues were dissected and weighed.

### 1.11 Statistical and analytical methods

The data in vitro are shown as mean ± SD. The data in vivo are shown as mean ± SEM. We did the statistical analysis using GraphPad Prism 6.0. Using a student’s t test for the difference analysis between the two groups. The difference among more than two groups using One-way ANOVA test. P < 0.05 was considered as statistically significant.

## 2. Results

Cervical cancer is a common malignant tumor in women. Understanding the pathogenesis of cervical cancer, looking for potential prognostic and targeted treatment indicators is of great significance to improve the subsistence rate of patients with CC. Lnc RNA MST1P2 has been reported increased in CC, but its mechanism in CC has not been clarified. In the present study, we conducted a series of in vitro and in vivo assays, aimed to explore the molecular mechanism of lncRNA MST1P2 in CC cells and the function in nude mice transplanted tumor, to lay a theoretical foundation for possible clinical application in the future.

### 2.1 Lnc MST1P2 down-regulation inhibited human CC malignancy

**Figure 1.**
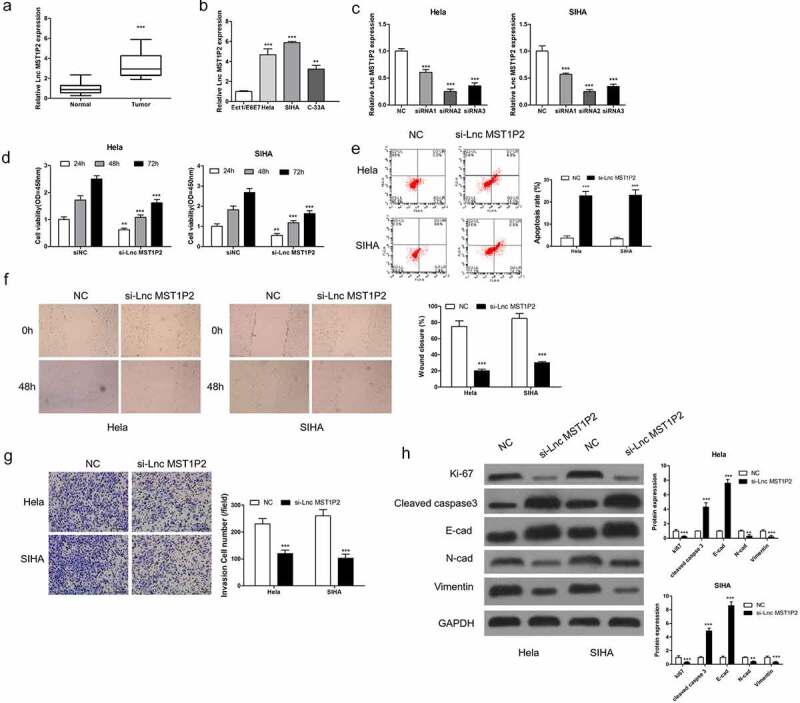
Lnc MST1P2 down-regulation inhibited human CC malignancy. (a) RT-qPCR was used to analyze Lnc MST1P2 level in CC tissues. (b) RT-qPCR analysis of Lnc MST1P2 expression in three CC cell lines and one normal cell line Ect1/E6E7. (c) Hela and SIHA cells were transfection with si-Lnc MST1P2 lentivirus, RT-qPCR analysis of si-Lnc MST1P2 level. (d) cell proliferation detected by CCK-8 assay. (e) Representative results of apoptosis assay. (f) cell migration detection. (g) cell invasion detection. (h) Western blot analysis of protein level. **P < 0.01, ***P < 0.001

RT-qPCR analysis was carried out on 40 pairs of CC samples and non-tumor tissues to evaluate Lnc MST1P2 expression level. As shown in Figure 1(a), Lnc MST1P2 was obviously increased in CC tissues in comparison with non-tumor tissues. Lnc MST1P2 levels in normal cervical epithelial cells Ect1/E6E7 and three CC cell lines (Hela, SIHA, C-33A) were also analyzed, as shown in Figure 1(b), Lnc MST1P2 expressed higher in the three kind of CC cell lines than that in Ect1/E6E7 cells (Figure 1(b)). The function of Lnc MST1P2 in CC malignancy was then explored. Hela and SIHA cells, which have high Lnc MST1P2 level, were transfection with si-Lnc MST1P2 or siNC. RT-qPCR analysis showed that Lnc MST1P2 was down-regulated in cells transfection with si-Lnc MST1P2 (Figure 1(c)). CCK8 analysis, cell apoptosis assay, cell migration and invasion assays, and western blot analysis were carried out to confirm the specific effects of lnc MST1P2 in CC. It turns out, Lnc MST1P2 knockdown dramatically repressed cell growth and cell metastasis (Figure 1(d–h)). In addition, Lnc MST1P2 knockdown inhibited metastasis (E-cad, N-cad and Vimentin) and proliferation (ki-67) related protein expression and induced apoptosis related protein expression (cleaved caspase 3) (Figure 1(e)). Lnc MST1P2 might serve as a tumor oncogene in CC.

### 2.2 Lnc MST1P2 targeting miR-133b in Hela and SIHA cells

**Figure 2.**
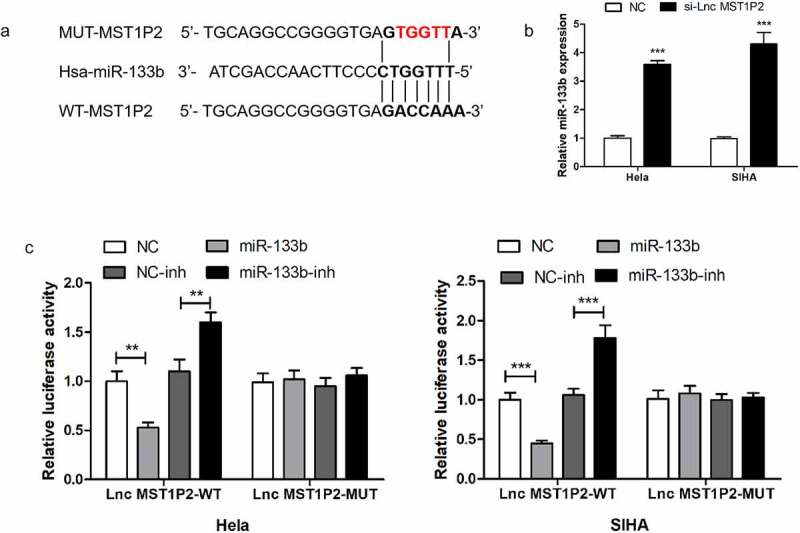
Lnc MST1P2 targeting miR-133b in Hela and SIHA cells. (a) Identification of miR-133b as a binding target of Lnc MST1P2. (b) detection of miR-133b level. GAPDG was used as an internal control. (c) Luciferase activity in Hela and SIHA cells. **P < 0.01, ***P < 0.001

MiR-133b was forecasted to bind with lnc MST1P2 directly in HELA and SIHA cells (Figure 2(a)). Then the luciferase reporter analysis was established to confirm it. As shown in Figure 2(c), luciferase activities were strikingly weakened in which cotransfection with the WT-lnc MST1P2 and miR-133b. RT-qPCR shown that si-lnc MST1P2 transfection remarkably enhance miR-133b level in Hela and SIHA cells (Figure 2(b)). Our results confirmed that miR-133b was a binding target of lnc MST1P2.

### 2.3 miR-133b inhibited CC cell progression

**Figure 3.**
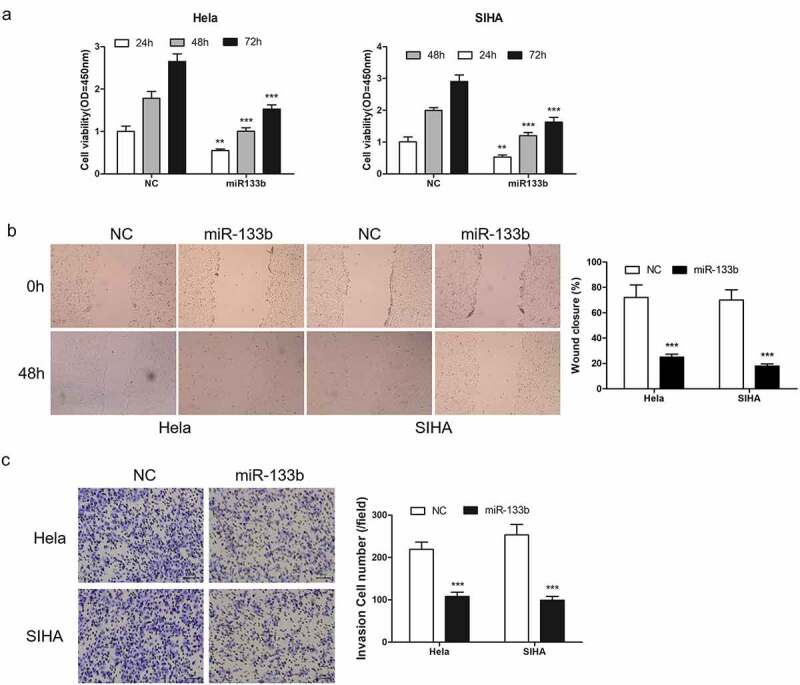
miR-133b overexpressed inhibited CC cell proliferation, cell migration and cell invasion. Cells were transfection in miR-133b mimics and control (NC). (a) detection of cell proliferation. (b) detect of cell migration. (c) detection of cell invasion. ***P < 0.001

The function of miR-133b in CC progression was then investigated. Hela and SIHA cells was transfected with miR-133b to increase miR-133b. Using CCK8 analysis detected the cell growth. The results showed that up-regulated expression of miR-133b obviously reduced the proliferation of cells (Figure 3(a)). Cell migration analysis (Figure 3(b)) and Transwell analysis (Figure 3(c)) confirmed that miR-133b effectively inhibited cell metastasis.

### 2.4 miR-133b-inh reverses anti-tumor effects of si-Lnc MST1P2 in Hela and SIHA cells

**Figure 4.**
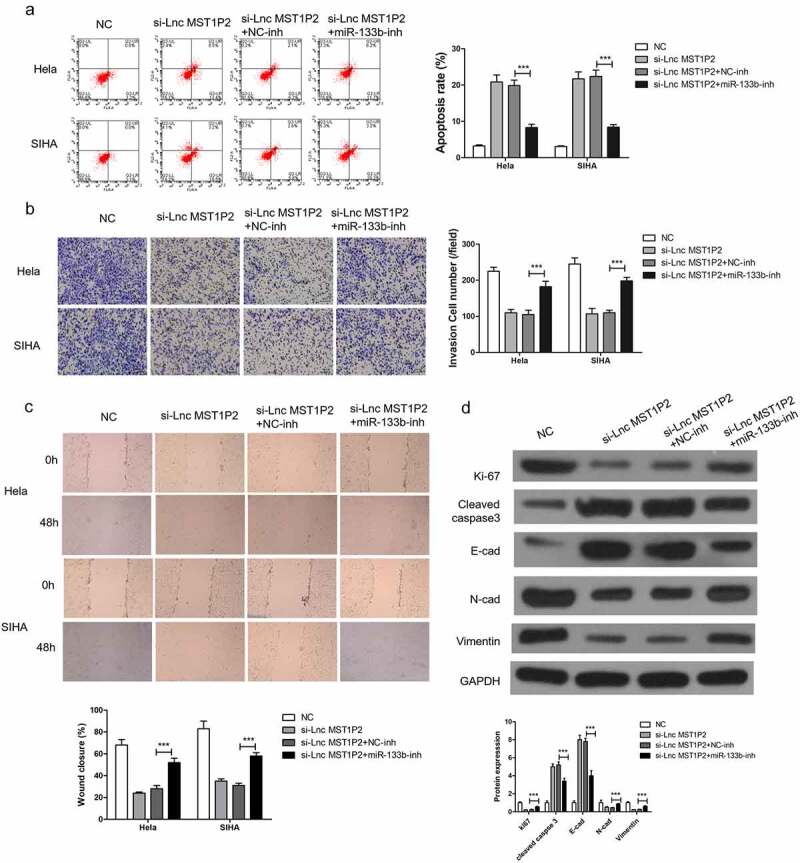
miR-133b inhibition reverses anti-tumor roles of si-Lnc MST1P2 in Hela and SIHA cells. Cells were co-transfection in si-Lnc MST1P2 and miR-133b inhibitor. (a) detection of cell apoptosis. (b) detection of cell invasion. (c) detection of cell migration. (d) Western blot analysis of protein level. ***P < 0.001

To study the function relevance of Lnc MST1P2 targeting miR-133b in Hela and SIHA cells, miR-133b-inh was transferred in Hela and SIHA cells. miR-133b-inh significantly restored si-Lnc MST1P2-reduced cell migration and cell invasion and si-Lnc MST1P2-induced cell apoptosis in Hela and SIHA cells (Figure 4(a–c)), moreover, miR-133b-inh inhibited si-Lnc MST1P2-reduced metastasis (E-cad, N-cad and Vimentin) and proliferation (ki-67) related protein expression and induced apoptosis related protein expression (cleaved caspase 3) (Figure 4(d)). These results indicated that Lnc MST1P2 promotes CC progression by binding with miR-133b.

### 2.5 miR-133b-inh reverses anti-tumor effects of si-Lnc MST1P2 in vivo

**Figure 5.**
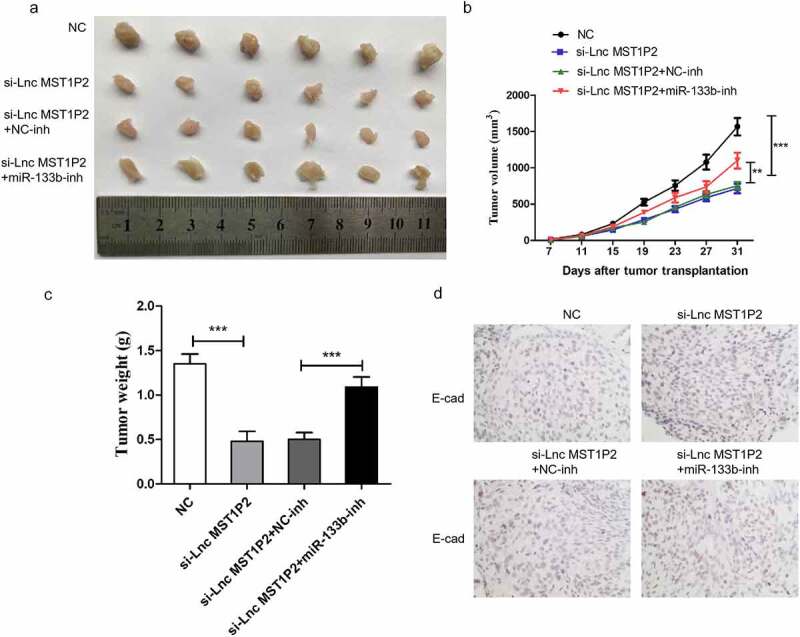
Lnc MST1P2 induces CC tumorigenicity in vivo by suppressing miR-133b. (a) Represented images of tumors. (b) Tumor volumes (c) Tumor weights. (d) E-cadherin expression level in tumors. **P < 0.01, ***P < 0.001

Hela cells was cotransfected with si-Lnc MST1P2 or si-NC and miR-133b-inh or miR-NC, the cells were then used to construct subcutaneous tumor model in nude mice. Tumor growth in si-Lnc MST1P2 group was notebaly slower than that in si-NC group (Figure 5(a)). Lnc MST1P2 downregulation presented smaller tumor size and weight than control group (Figure 5(b,c)). We measured the levels of E-cad in the tumor tissue. Compared with si-NC group, si-Lnc MST1P2group had up-regulated E-cad expression (Figure 5(d)). MiR-133b-inh could obviolusly reverse anti-tumor effect of si-Lnc MST1P2 in vivo, which indicating Lnc MST1P2 promote CC tumorigenicity by suppressing miR-133b.

## 3. Discussion

In the present study, we shown that Lnc MST1P2 was apparently overexpression in CC tissues as well as cells when comparerd to the paired non-tumor tissues and normal cells. Furthermore, to investigate the undelying mechanism and clinical significance of Lnc MST1P2 in the development of CC, two stable HeLa and SIHA cell lines with Lnc MST1P2 interference expression was constructed. low expression of Lnc MST1P2 in HeLa and SIHA cells could inhibit cell viability, cell invasion and cell migration. Low expression of Lnc MST1P2 in vivo can inhibit tumor growth in nude mice. which indicated that Lnc MST1P2 might act as an oncogene in CC.

It has been proved Lnc MST1P2 making a significant contribution to the development of tumor. Lnc MST1P2 expression was significantly up regulated after tumor radiotherapy. Lnc MST1P2 was also enhanced in cisplatin-resistant bladder cancer cells and regulates the drug-resistance of cells by miR-133b [15]. In addition, we used Starbase calculated a complementary binding site between mir-133b and Lnc MST1P2. luciferase reporter analysis and RT-qPCR further showed that Lnc MST1P2 negatively regulated mir-133b in HeLa and SIHA cells. Mir-133b function in a variety of cancers differently. MiR-133b suppressed gastric cancer cell growth and invasion by inducing fibrillin 1 [22]. miR-133b have been proved to serves as a tumor inhibitor in glioma [23]. miR-133b has been proved that down-regulation in CC [21].Our paper also confirmed that miR-133b was downregulation in HeLa and SIHA cells. Which was consistent with previous studies. We also proved miR-133b overexpression dramatically inhibited cell growth, cell migration and cell invasion.

Further, miR-133b-inhibition significantly attenuate anti-cancer function of si-Lnc MST1P2 both in vitro and in vivo, which verified that Lnc MST1P2 directly targeting miR-133b in CC. Given that miR-133b has a key role in repressing human tumors malignancy, targeting miR-133b may be an effective new therapy strategy for the prevention of CC progression.

## 4. Conclusions

Our study elucidates that Lnc MST1P2 is up-regulation and miR-133b is down regulated in CC. Lnc MST1P2 targets miR-133b and regulates Hela and SIHA cell proliferation and apoptosis. Lnc MST1P2 may be a potential molecular target of CC.

## Supplementary Material

Supplemental Material

## Highlights

- LncRNA MST1P2 is obviously overexpressed in cervical cancer tissues and cells.
- LncRNA MST1P2 promotes cervical cancer tumorigenesis both in vitro and in vivo.
- LncRNA MST1P2 serves as a cervical cancer oncogene by sponging with miR-133b.
