# Supplementary figures and images for: Long noncoding RNA MST1P2 promotes cervical cancer progression by sponging with microRNA miR-133b
Source: Bioengineered. 2021 May 25;12(1):1851–60. doi: 10.1080/21655979.2021.1921550 (PMC8806230; doi:10.1080/21655979.2021.1921550)

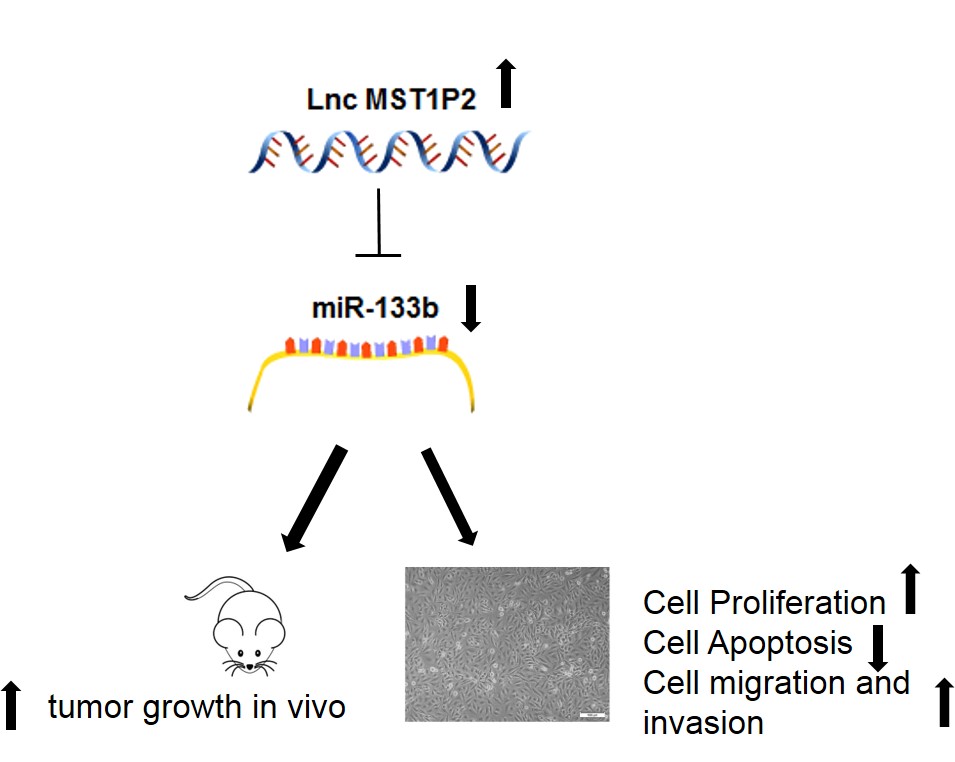

Supplement: Supplemental Material [file KBIE_A_1921550_SM8601.jpg]
